# A novel mode of cytokinesis without cell-substratum adhesion

**DOI:** 10.1038/s41598-017-17477-w

**Published:** 2017-12-18

**Authors:** Risa Taira, Shigehiko Yumura

**Affiliations:** Department of Functional Molecular Biology, Graduate School of Medicine, Yamaguchi University, Yamaguchi, 753-8512 Japan

## Abstract

Cytokinesis is a final step in cell division. *Dictyostelium* cells, a model organism for the study of cytokinesis, have multiple modes, denoted cytokinesis A, B, C, and D. All these modes have been mainly investigated using cells adhering to the substratum although they can grow in shaking suspension culture. Here, we observed how cells divide without adhering to the substratum using a new non-adhesive material. These detached cells formed the cleavage furrow but eventually failed in the final abscission. Thus, the cells cannot divide without adhesion, suggesting that they cannot divide only through the conventional cytokinesis A. However, in a long-term culture, the detached cells adhered each other to form multicellular aggregates and divided properly in these aggregates. Myosin II-null cells also formed such aggregates but could not divide in the aggregates. Several lines of experiments using mutant cells showed that the process of cytokinesis in multicellular aggregates is a novel mode utilizing a confined space in the aggregate in a myosin II-dependent manner. These results shed light on a poorly characterized mechanism of cytokinesis in multicellular spheroids or tissues. We propose to redefine and classify multiple modes of cytokinesis.

## Introduction

Cytokinesis is the final step in cell division and involves the division of the cytoplasm of the parental cell into two daughter cells. Cytokinesis failure results in the multinucleation of cells and often causes tumors or cancers in the human body^1,2^. In animal cells and lower organisms, such as *Dictyostelium* cells, constriction of the contractile ring, which is mainly composed of actin and myosin filaments, has been considered to pinch the cell into two^3,4^. Previous studies have elucidated the existence of several different cytokinesis mechanisms from this ‘purse string model’. Myosin II-null *Dictyostelium* cells cannot actively constrict the cleavage furrow and thus become multinucleated in shaking culture. However, these cells can divide on the substratum after adhesion, and this process has been termed “attachment-assisted mitotic cleavage”^5^. More recently, the conventional process of cytokinesis in wild-type cells was denoted “cytokinesis A”, and that of myosin II-null cells was termed “cytokinesis B”^6^.

The placement of large multinucleated myosin II-null cells, which were generated by culturing in suspension, on a substratum results in their adherence to the substratum and subsequent division into multiple fragments, finally each containing one nucleus; this process is called “traction-mediated cytofission”^7^. This cytofission occurs in a manner unrelated to cell cycle progression; in fact, these multinucleated cells often divide during interphase. This process was later termed “cytokinesis C” to distinguish it from cytokinesis A and B^8^. Recent studies have provided increasing evidence that higher-animal cells can divide also by cytokinesis B and C^9,10^.

In addition to cytokinesis A, B, and C, a novel mode (cytokinesis D) was identified in *Entamoeba* cells; these cells have been found to divide with assistance from neighbor cells, which act as midwifes^11^. Dividing cells emit a chemoattractant to attract neighboring cells from the equatorial region, and this chemoattractant induces the neighboring cells to move over the cleavage furrow to aid the scission process. The cytokinesis D process of *Dictyostelium* cells has been reported to require the ß-subunit of trimeric G proteins, which is essential for chemotaxis^12^.

In previous studies, cytokinesis has been mainly observed in cells adhering to the substratum. In contrast, unanchored fibroblasts cannot complete cytokinesis^13^. *Dictyostelium* mutants deficient in talin, paxillin, vinculin or sadA, which show defects in cell-substratum adhesion, frequently fail in cytokinesis^14–17^, suggesting the importance of adhesion during cell division.

In this study, we made the substratum surface non-adhesive using a new coating material to observe how cells divide without adhesion to the substratum. Surprisingly, these detached cells formed the cleavage furrow but ultimately failed to separate and became multinucleated, suggesting that they cannot divide only through the conventional cytokinesis A without adhering to the substratum. Interestingly, the long-term culture of these cells in this “detached condition” resulted in the formation of multicellular aggregates. These cells could divide normally in these aggregates and multiply at a rate similar to those on the substratum. From several lines of experiments using mutant cells, we concluded that the process of cell division in multicellular aggregates is a novel mode for cytokinesis. These results shed light on a poorly characterized mechanism of cytokinesis in multicellular spheroids or tissues. Finally, we proposed to redefine and classify multiple modes of cytokinesis.

## Results

### Cells cannot divide without adhering to the substratum

**Figure 1:**
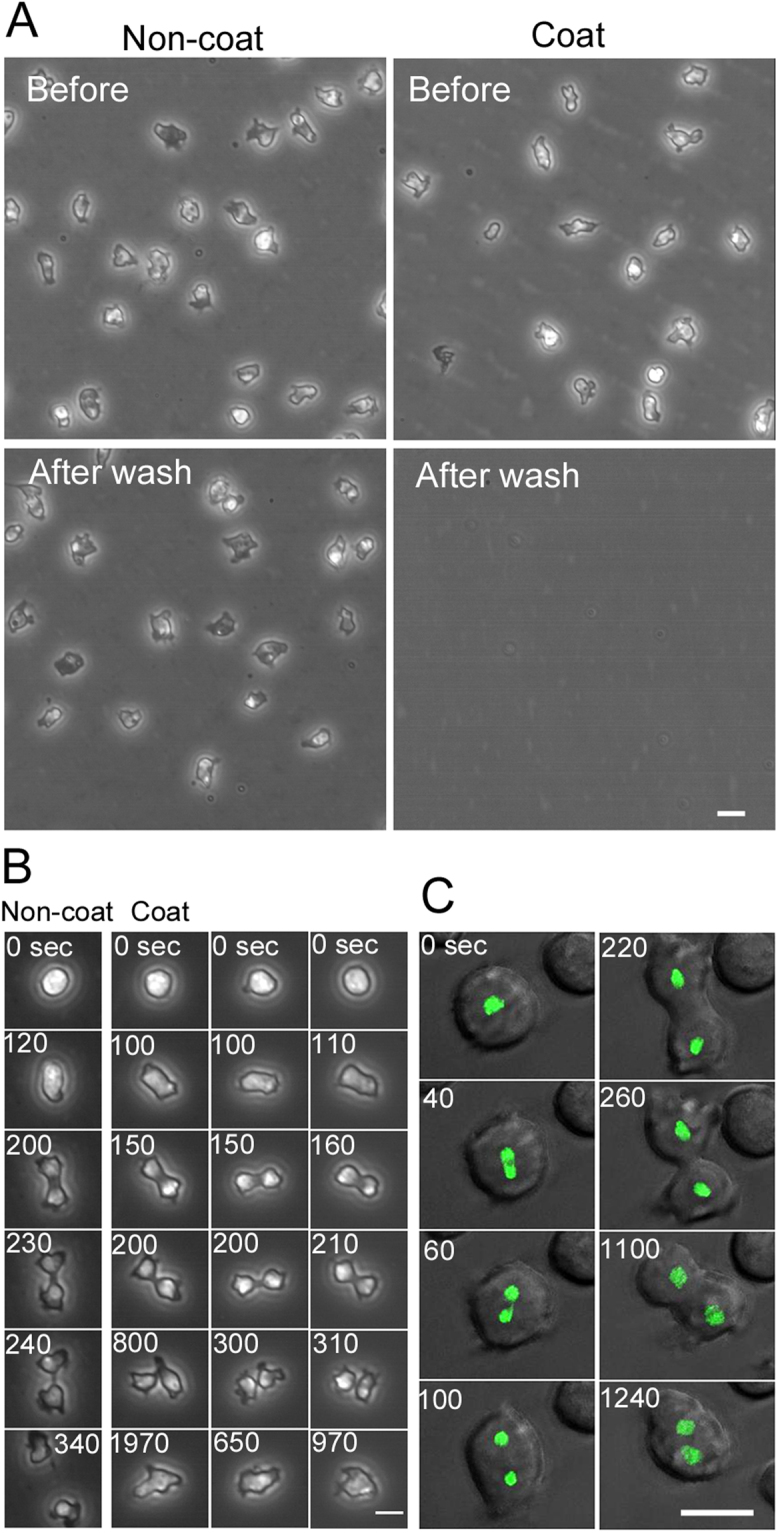
Cells cannot divide without adhering to the substratum. (**A**) When *Dictyostelium* cells were placed on the coverslip coated with Lipidure, the cells could not adhere to the coverslip and just showed Brownian movement. To demonstrate the detachment of the cells, 20 min after the cells were placed and settled on the coated coverslip, all of the cells were washed off by medium exchange using a fine-tip aspirator. In contrast, most of the cells remained on the non-coated coverslip after the medium was exchanged. The field of view was the same (Before and After wash). (**B**) Typical time courses of cell division on non-coated (left column) and coated coverslips (three right columns) obtained by phase-contrast microscopy. Cells entering the mitotic phase became spherical in shape and subsequently formed the cleavage furrow. However, none of the observed cells could split into two daughter cells under the detached condition (Coat), although the cells on the non-coated substratum successfully underwent cell division (Non-coat). The time of furrow closing was not significantly different between non-coated and coated conditions (161.3 ± 31.9 sec and 165.2 ± 31.0 sec, respectively, n = 55). However, the abscission time (from thread formation to final cutting) was about 60 sec (59.3 ± 18.9 sec, n = 55) in non-coated condition, but all examined cells on coated surface finally failed in abscission and became binucleated by fusion. In latter case, they took longer time until fusion (774.3 ± 352.7 sec, n = 55). We observed 55 cells in three independent experiments both in coated and non-coated conditions, and confirmed the results shown in Panel B. (**C**) Typical time course of failure in cytokinesis of a cell expressing GFP-histone (green), a nuclear marker, on the coated coverslip. The cell finally became binucleated. Bars, 10 µm.

To examine how cells divide without adhering to the substratum, the coverslip was coated with Lipidure, a new non-adhesive coating material, which has the same structure as the phosphatidylcholine polar bases that form the cell membrane^18^. The placement of *Dictyostelium* cells on the coated coverslip resulted in none of the cells adhering to the coverslip. To demonstrate the effectiveness of the coating, 20 minutes after the cells were placed and settled on the coated coverslip, all of the cells were washed off by mildly exchanging the medium (right panels in Fig. 1A). In contrast, most of the cells remained on the non-coated coverslip after the medium was exchanged (left panels in Fig. 1A).

Dividing cells were observed under this “detached condition”. Although the position of the cells always changed due to Brownian motion, the cells could not migrate but did change their shape by extending pseudopods. The cells became spherical at the beginning of the mitotic phase, formed the cleavage furrow, and constricted it. However, all of observed cells failed in the final abscission (three right columns in Fig. 1B, n = 55). In contrast, all of observed cells on the non-coated substratum successfully underwent cell division (left column in Fig. 1B, n = 55). Figure 1C shows a typical failure in cytokinesis of a cell expressing GFP-histone, a marker of nucleus, on the coated substratum. The cell finally became binucleated, suggesting proper occurrence of the nuclear division. Cells cultured on a coverslip coated with poly-HEMA, another non-adhesive coating material, resulted in a similar failure in cytokinesis as those cultured on the Lipidure-coated coverslip.

Together, the results demonstrate that *Dictyostelium* cells cannot divide without adhering to the substratum. Although they have been thought to divide only by cytokinesis A in suspension culture, they must need an additional mechanism for abscission.

### Cells can divide in multicellular aggregates

**Figure 2:**
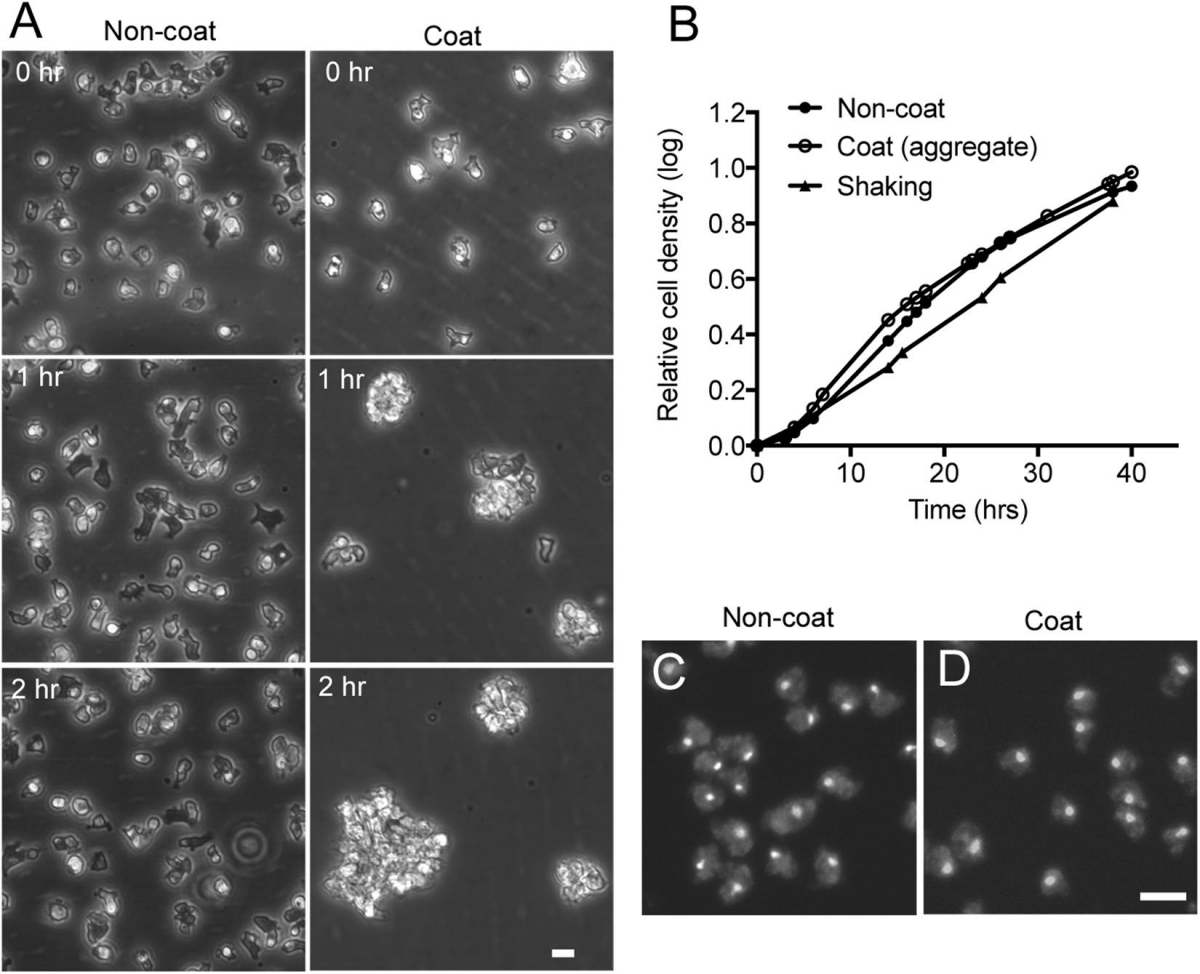
Cells can divide in multicellular aggregates. (**A**) When cells were cultured in the Lipidure-coated plastic dish, they gradually adhered to each other to form multicellular aggregates within 1–2 h (right column), whereas the cells adhered to the substratum separately on the non-coated surface (left column). (**B**) Cell growth rate under three different conditions: on a non-coated surface (closed circles), on a coated surface (cells formed multicellular aggregates, open circles), and in shaking culture (closed triangles). The cells grown in shaking culture were cultured in a conical flask at 150 rpm. A small aliquot of the cell culture was taken at indicated time, dissociated by pipetting, and then the cell density was counted by a hemacytometer. The growth rates were almost equal in all conditions. (**C** and **D**) Nuclei of the cells after culture in different conditions: on a non-coated surface (**C**) and on a coated surface (**D**). The cell aggregates were dissociated into single cells by pipetting, fixed with a fixative and then stained with DAPI for fluorescence microscopy. Note that most of the cells had a single nuclei in both conditions, suggesting that the cells divide properly in the multicellular aggregates. Bars, 10 µm.

**Figure 3:**
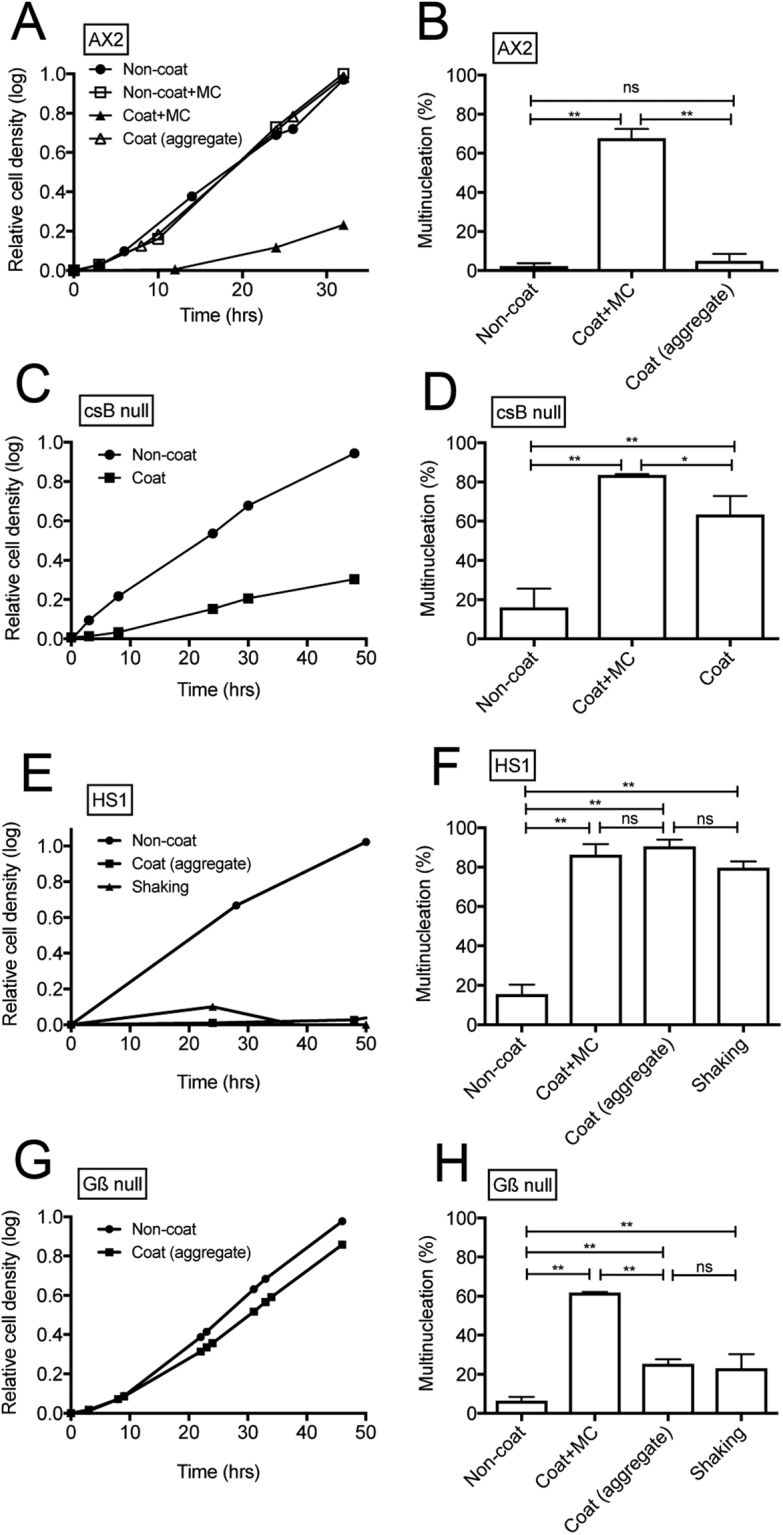
Cell growth and multinucleation of mutant cells in different culture conditions. (**A**) Cell growth of wild-type cells in the presence (closed triangles) and absence of methyl cellulose (MC, open triangles) in a Lipidure-coated dish. The growth of wild-type cells in the presence (open rectangles) and absence of MC (closed circles) in the non-coated dish was also plotted. Because the cells gradually formed aggregates even in the presence of MC, the formed aggregates were dispersed into single cells by pipetting every 5 h. (**B**) Frequency of multinucleation of wild-type cells (AX2) in different conditions: in a non-coated dish, in a coated dish containing MC, and in a coated dish (the cells formed multicellular aggregates). (**C**) Cell growth of csB-triple-mutant cells under different conditions, specifically in non-coated (closed circles) and coated (closed squares) dishes. (**D**) Frequency of multinucleation of csB-triple-mutant cells in different conditions: in a non-coated dish, in a coated dish containing MC, and in a coated dish. (**E**) Growth curves of myosin II-null cells at different culture conditions: in a non-coated dish (closed circles), in a coated dish (the cells formed multicellular aggregates, closed squares), and in shaking culture (closed triangles). (**F**) Frequency of multinucleation at different culture conditions; in a non-coated dish, in a coated dish, in a coated dish containing MC, and in shaking culture. Note that myosin II-null cells cannot multiply in the multicellular aggregates and became multinucleated. (**G**) Growth curves of Gß-null cells at different culture conditions: in a non-coated dish (closed circles) and in a coated dish (the cells form multicellular aggregates, closed squares). (**H**) Frequency of multinucleation under different culture conditions: in a non-coated dish, in a coated dish, in a coated dish containing MC and in shaking culture. For the multinucleation analysis (**B**,**D**,**F**, and **H**), the cells were stained with DAPI after 24 h of culture (more than 500 cells were counted for each condition in three independent experiments). Data are presented as mean ± SD and analyzed by one way ANOVA with Tukey’s multiple comparison test. *P ≤ 0.05; **P ≤ 0.001; ns, not significant, P > 0.05.

When cells were cultured in a Lipidure-coated plastic dish, they gradually adhered each other, resulting in the formation of multicellular aggregates within 1–2 h (Fig. 2A and Supplementary Video S1). To the best of our knowledge, this process has not been reported previously. Interestingly, the cells could multiply in the aggregates, because their growth rate was almost equal to that of cells cultured adhering to the substratum or in shaking condition (Fig. 2B). This finding also suggests that Lipidure coat does not have any harmful effect on cell division and growth. To examine the number of nuclei in each cell, the cells were fixed and stained with 4′, 6-diamidino-2-phenylindole (DAPI). Most of the cells had a single nucleus, suggesting that the cells divide properly in the multicellular aggregates (Figs 2D and 3B). Cells that were cultured adhering to the non-coated substratum, which served as a control, also had single nuclei (Fig. 2C). Placement of the multicellular aggregate on a non-coated coverslip resulted in the dispersion of the aggregate into individual migrating cells within 10 min (Supplementary Fig. S1), suggesting that the aggregate is not formed by directly connecting each of the cells by Lipidure, which might be released from the coated substratum.

To examine whether cells become multinucleated when the formation of the multicellular aggregate was inhibited in long-term culture, the cells were cultured in the coated dish with medium containing methyl cellulose (MC) to increase the viscosity of the culture medium, which resulted in significant suppression of Brownian motion and thereby reduced cell aggregation (Supplementary Fig. S2A). In another study, mutant cells deficient in three genes encoding cell-cell adhesion contact site B (csB) proteins (*csbA*, *csbB*, and *csbC*)^19^ were cultured in the coated dish. The formation of cell aggregates was significantly suppressed (Supplementary Fig. S2B). Supplementary Fig. S2C,D show typical DAPI-stained fluorescence images showing that the cells became severely multinucleated in both cases. Figure 3A shows the growth curves of wild-type cells (AX2) on the coated and non-coated surface in the presence and absence of MC. Their growth on the coated surface was significantly suppressed in the presence of MC compared with on non-coated surface. Figure 3C shows the growth curves of csB-triple-mutant cells on the non-coated and coated surface. Their growth was also significantly suppressed on the coated surface. Figure 3B,D show a summary of multinucleation in different conditions. In both conditions aiming to reduce cell aggregation (in the presence of MC or using csB-mutant cells), the cells became multinucleated. In the presence of MC, the csB-mutant cells became more severely multinucleated (Fig. 3D).

Together, wild-type cells cannot divide without adhering to the substratum and can divide at a proper rate if they form multicellular aggregates.

### Mode of cytokinesis in multicellular aggregates

Several modes of cytokinesis in addition to cytokinesis A, specifically cytokinesis B, C, and D, have been reported in *Dictyostelium* cells. The mode of cytokinesis that contributes to cytokinesis in multicellular aggregates was subsequently examined. One possible and simple explanation for cytokinesis in multicellular aggregates is that cells exert traction force against the surface of other cells instead of the substratum in a manner similar to cytokinesis B and C. Cytokinesis B and C do not require myosin II. The culture of myosin II-null cells (HS1) in a Lipidure-coated dish resulted in the formation of multicellular aggregates, similar to the effect on wild-type cells (Supplementary Fig. S3A,B). However, the cell growth was significantly suppressed and they became multinucleated in the aggregates (Fig. 3E,F), suggesting that myosin II is required for cytokinesis in multicellular aggregates. Thus, the mode of cytokinesis in aggregates cannot be explained by cytokinesis B or C, which are independent of myosin II.

Another possibility is that other cells are attracted by chemotaxis to the cleavage furrow and aid the process of cell division by crossing over the furrow (cytokinesis D). Nagasaki and Uyeda (2008) previously reported that cells deficient in the ß-subunit of trimeric G protein (Gß-null cells) show a significantly reduced rate of cytokinesis D (approximately 84% reduction)^12^. The culture of Gß-null cells in the Lipidure-coated dish resulted in the formation of multicellular aggregates, similar to wild-type cells (Supplementary Fig. S3C,D), and normal cell growth without a severe deficiency in cytokinesis in the aggregates (Fig. 3G,H), suggesting that the mode of cytokinesis in the aggregates does not depend on cytokinesis D.

Together, these results show that the cytokinesis in multicellular aggregates is a novel mode of cytokinesis that is dependent on myosin II.

### Observation of cell division in multicellular aggregates

**Figure 4:**
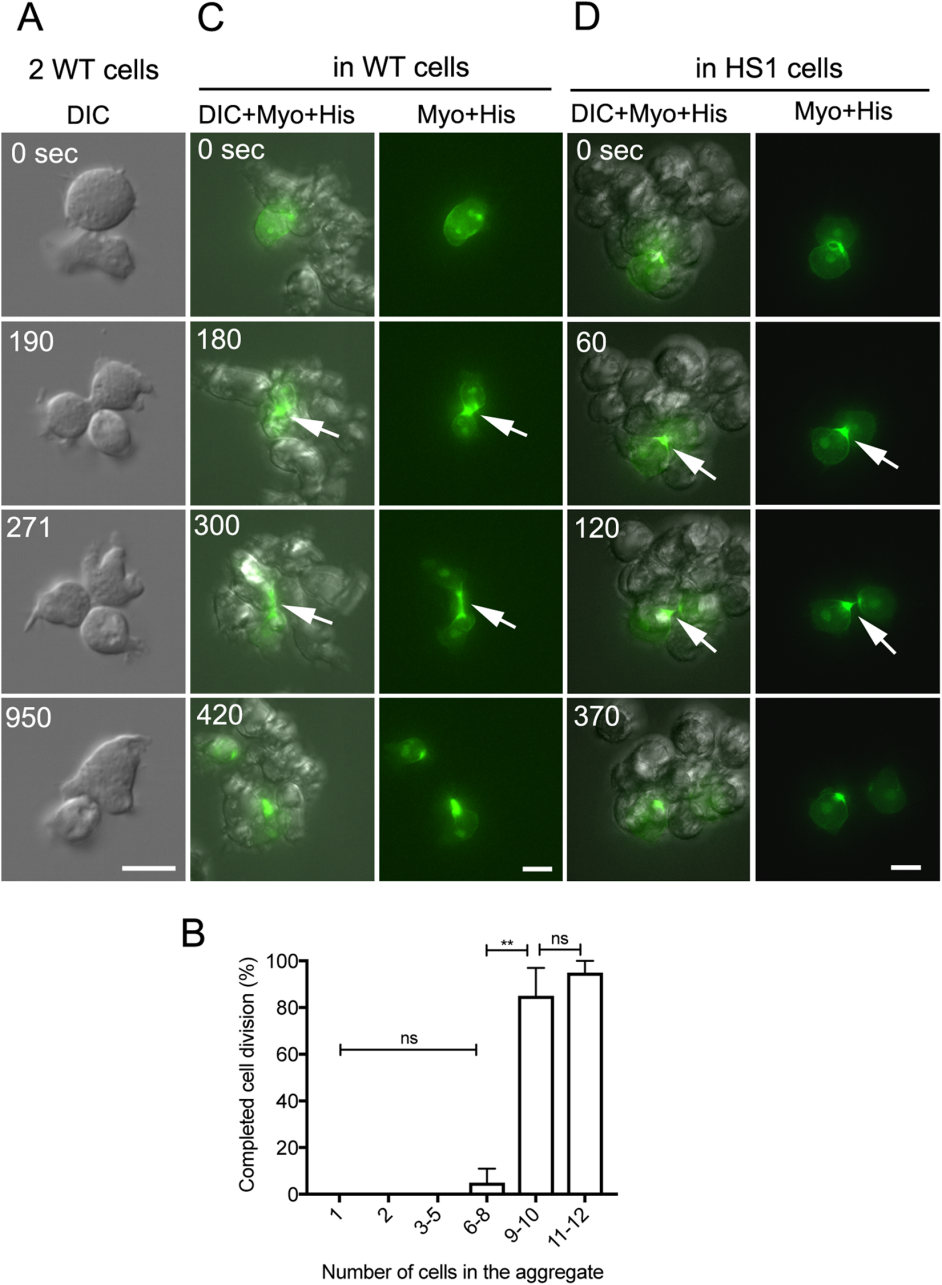
Observation of cell division in multicellular aggregates. (**A**) When the dividing cell adhered to another single cell, it formed the cleavage furrow but eventually failed in cytokinesis. (**B**) Frequency of successfully completed cell division depending on the number of cells composed of the aggregate. Data are presented as mean ± SD in five independent experiments (n = 30 dividing cells, each). **P ≤ 0.001; ns, not significant, P > 0.05. (**C**) Typical time course of wild-type cell division in a multicellular aggregate. Wild-type cells simultaneously expressing GFP-myosin II and GFP-histone were mixed with wild-type cells not expressing GFPs at a ratio of 1:100. The fluorescence images were acquired by z-sectioning at 1-μm intervals over time under a sectioning fluorescence microscope and then reconstituted into three-dimensional images. Merged images of GFP-myosin II, GFP-histone, and DIC (left column) and of GFP-myosin II and GFP-histone (right column) are shown. Note that the fluorescent cell completed cytokinesis and that myosin II accumulated at the furrow (arrows) in the multicellular aggregate (composed of 9 cells). (**D**) Typical time course of wild-type cell division in a multicellular aggregate of myosin II-null cells (composed of 10 cells). Wild-type cells simultaneously expressing GFP-myosin II and GFP-histone were mixed with myosin II-null cells not expressing GFPs were mixed at a ratio of 1:100. Merged images of GFP-myosin II, GFP-histone, and DIC (left column) and of GFP-myosin II and GFP-histone (right column) are shown. Note that the fluorescent cell completed cytokinesis and that myosin II accumulated at the furrow region (arrows). Bars, 10 µm.

The above-mentioned observations show that cells can divide as long as they adhere to other cells, even when detached from the substratum. However, the adherence of a dividing cell to a single cell resulted in a failure in cytokinesis (Fig. 4A). How many cells are required for the cytokinesis in the aggregate? Fig. 4B shows a summary of the frequency of successfully completed cell division depending on the number of cells in the aggregate, suggesting that at least more than 9–10 cells is required for the successful cytokinesis. Presumably, the cells need to be surrounded by several cells to divide.

To directly observe the process of cell division in multicellular aggregates, cells simultaneously expressing GFP-myosin II and GFP-histone were mixed with cells not expressing GFP-fusion proteins at a ratio of 1:100. The fluorescence images were acquired by z-sectioning at 1-μm intervals over time under a sectioning fluorescence microscope, and three-dimensional images were reconstituted (Fig. 4C). The dividing cells in the multicellular aggregates finally succeeded in cytokinesis after nucleus division. Note that myosin II accumulated in the furrow region (arrows in Fig. 4C).

As described in the previous section, the experiments using myosin II null (HS1) cells showed that the cytokinesis in multicellular aggregates requires myosin II. The cells surrounding the dividing cell might require myosin II to provide pressing or pushing power to the diving cells to aid cell division. To clarify this point, wild-type cells simultaneously expressing GFP-myosin II and GFP-histone were mixed with HS1 cells not expressing GFP-fusion proteins at a ratio of 1:100 (Fig. 4D). In aggregates with HS1 cells, wild-type cells could complete cytokinesis. We confirmed the same results in multiple experiments (n = 24). Therefore, myosin II is required in the dividing cell but not in the surrounding cells for the successful cytokinesis.

### Cells can divide in a confined environment without any specific adhesion

How do cells divide in multicellular aggregates? Recent studies have shown that animal cells migrate in a different mode in a 3D confined environment^20^. For example, glioma cells migrate independently of myosin II on a two-dimensional surface, but myosin II is required for squeezing the cell body through a densely packed 3D microenvironment to invade in the brain^21^. Integrin-deficient leukocytes cannot adhere to the substrate but can migrate in a 3D collagen gel^22^. Carcinomsarcoma (Walker 256) deficient in substratum-attachments cannot migrate on a 2D surface but can migrate using friction in a confined environment, such as that observed in a microfluidic chamber^23^.

**Figure 5:**
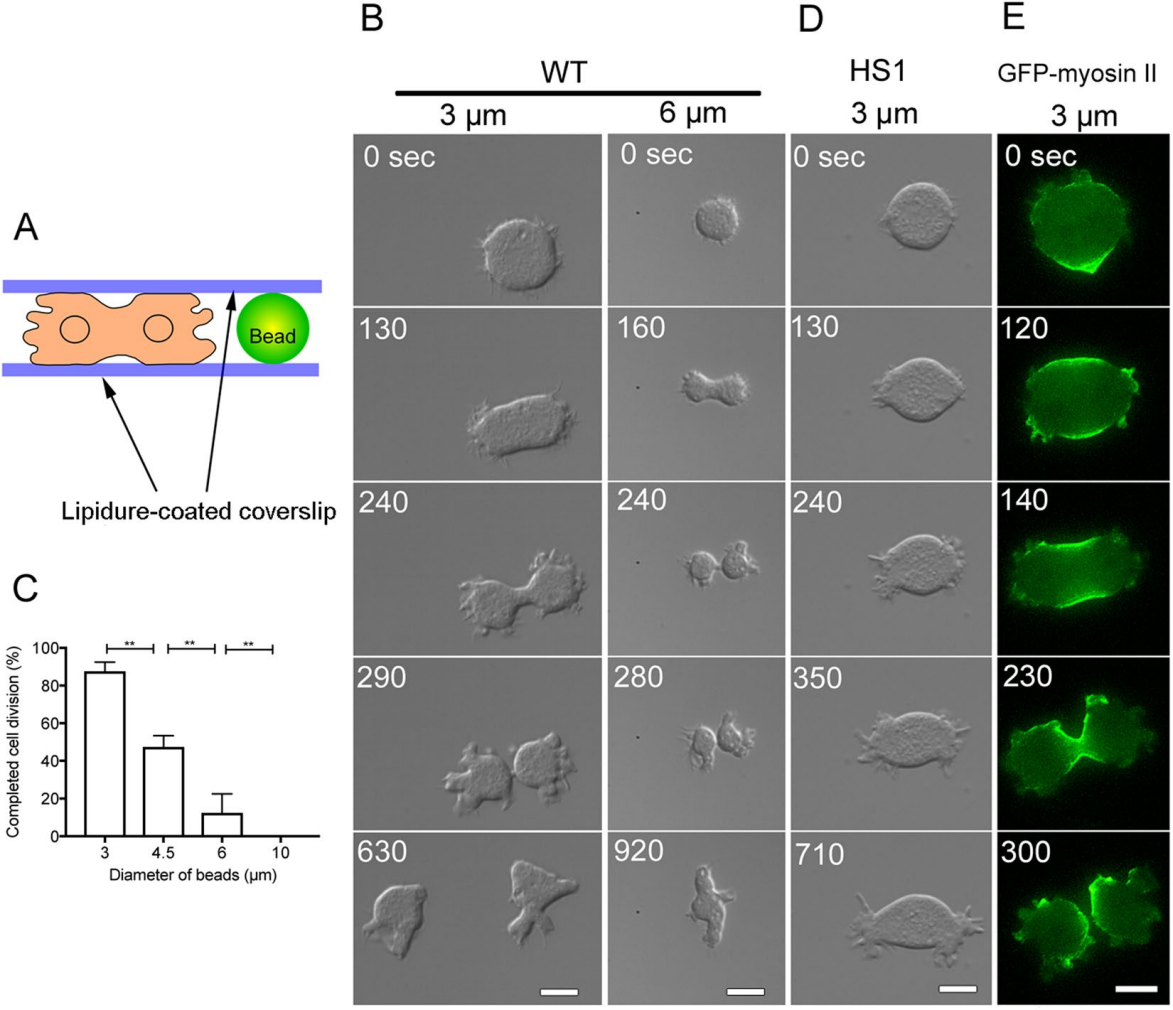
Cells can divide in a confined environment without any specific adhesion. (**A**) Cells were sandwiched between two Lipidure-coated coverslips. To obtain a space with a defined width, polystyrene beads (3, 4.5, 6, or 10 µm in diameter) were mixed with the cells and placed between two Lipidure-coated coverslips. (**B**) Typical time course of wild-type cell division in spaces with a width of 3 µm and 6 µm. In a 3-µm-wide space, the cell completed cytokinesis (left column), whereas the cells failed in a space with a width of 6 µm (right column). (**C**) A summary of the frequency of successfully completed cell division in space with various widths (beads sizes) between the two coverslips. Data are presented as mean ± SD in three independent experiments (n = 20 cells, each). **P ≤ 0.001; ns, not significant, P > 0.05. (**D**) Typical time course of failure of a myosin II-null cell in a space with a width of 3 µm. (**E**) Typical time course of myosin II-null cells expressing GFP-myosin II in a space with a 3-µm width between the two coverslips. Note that myosin II accumulated at the cleavage furrow during cytokinesis. Bars, 10 µm.

To investigate whether cells can divide utilizing the confined space of a multicellular aggregate, the confined environment was mimicked by placing cells between two Lipidure-coated coverslips. The width (depth) between the two coverslips was set by mixing the cells with microspheres of one of indicated sizes (3, 4.5, 6, or 10 µm in diameter) for spacer (Fig. 5A). Incidentally, the average cell diameter found when the cells became spherical during the mitotic phase was approximately 9–10 µm. Interestingly, wild-type cells (WT) could divide in the confined space. Most of the flattened cells with a width of 3 µm completed cell division, but those with a width of 6 µm presented a failure in cytokinesis (Fig. 5B). Figure 5C shows a summary of frequency of successfully completed cell division in space with various widths, indicating that cells can complete cell division in the confined space. In contrast, myosin II-null cells (HS1) always failed in cytokinesis, even in a 3-µm-wide space (n = 20, Fig. 5D). Placement of HS1 cells expressing GFP-myosin II in the confined space confirmed that myosin II accumulated in the furrow region (n = 20, Fig. 5E).

It appears likely that cells can divide utilizing confined environments within the multicellular aggregates, maybe by a friction-mediated mechanism, similar to the cells in a confined space between the coverslips.

### Shearing force is required for cell division in shaking culture

**Figure 6:**
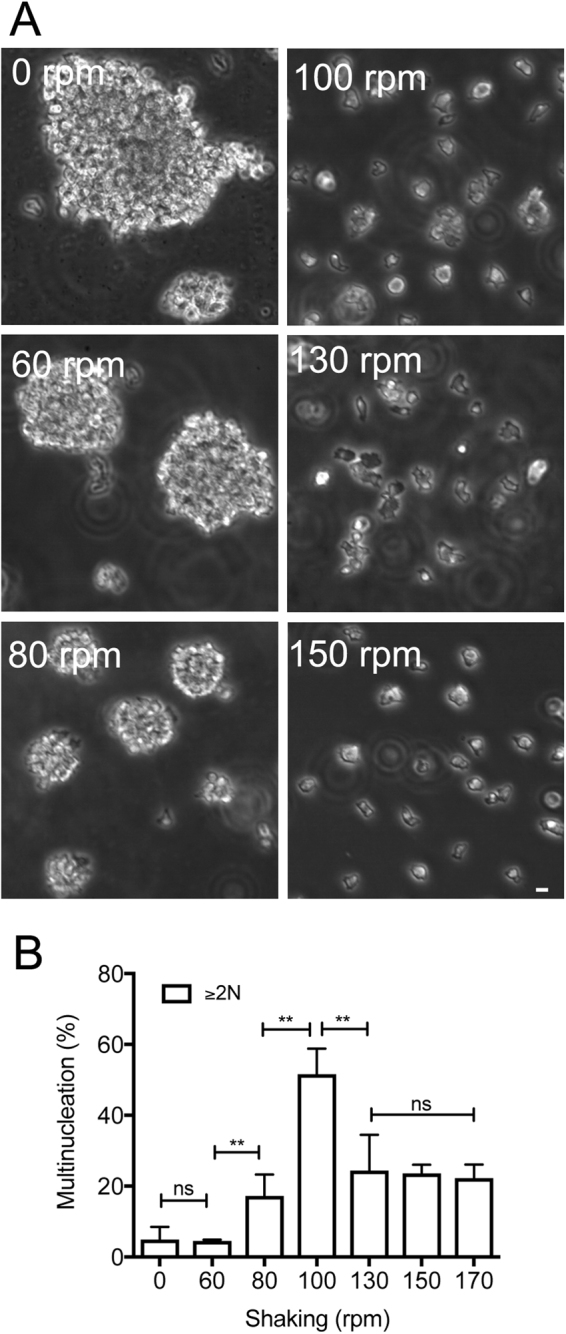
Shearing force is required for cell division in shaking culture. (**A**) Phase-contrast microscopy images of wild-type cells cultured for 24 h in shaking culture at 60, 80, 100, 130, and 150 rpm. As a control, the cells were cultured in a Lipidure-coated dish without shaking (0 rpm). Note that large multicellular aggregates formed at speeds lower than 80 rpm. (**B**) Frequency of multinucleation of the cells cultured at different shaking speeds. The cells were stained with DAPI (more than 500 cells were counted for each shaking condition in three independent experiments). At lower speed (<60 rpm), the cells formed multicellular aggregates, and most of cells remained mononucleated. At moderate speed (100 rpm), most of the cells were separated and became multinucleated (51.3 ± 7.2%, n = 1,500). At the standard speed (150 rpm) and higher, all of the cells were separated and showed less multinucleation (~20%). Data are presented as mean ± SD. **P ≤ 0.001; ns, not significant, P > 0.05. Bar, 10 µm.

*Dictyostelium* cells can be cultured in shaking conditions as well as adhering to the surface of culture dishes. As described above, because the cells cannot divide without adhering to the substratum, the shearing force in shaking culture might aid cell division. To confirm this possibility, the cells were cultured at different shaking speeds. Incidentally, in the standard cell culture protocol used in our laboratory, the shaking speed is set to 150 rpm. Figure 6A shows the degree of cell aggregation observed at different shaking speeds (rpm), which indicates that the aggregate size is decreased and that most of the cells were separated at speeds higher than 100 rpm. Figure 6B shows a summary of the number of nuclei in the cells observed at different shaking speeds. At a lower speed (<60 rpm), the cells formed multicellular aggregates and remained mononucleated. At a moderate speed (100 rpm), most of the cells were separated and became multinucleated (51.3 ± 7.2%, n = 1,500 cells), whereas at the standard speed (150 rpm) and above, all of the cells were separated and showed less multinucleation (~20%). From these results, we conclude that the shearing force exerted by shaking aids cell division in shaking culture at the standard speed.

## Discussion

Cytokinesis A (purse-string model) in animal cells and lower organisms, such as *Dictyostelium* cells, has been considered a robust and common mechanism for the division of a mother cell into two daughter cells. The present study revealed that *Dictyostelium* cells cannot divide only by the conventional cytokinesis A. In the final step of cytokinesis, the connection between two daughters of a dividing *Dictyostelium* cell have no special structure, such as the ‘midbody’, which is known as a machinery for the final abscission in higher-animal dividing cells. Recent intensive studies have indicated that many components, such as Arf/Rab small GTPases, SNARE proteins, ESCRT-III, and membrane vesicles, accumulate at the midbody to form the abscission machinery in animal cells^24–26^. The reason why *Dictyostelium* cells cannot divide without adhering to the substratum might simply be the absence of a midbody. Instead, *Dictyostelium* cells might have multiple modes of cytokinesis, including the mode discovered in the present study. These cells might not acquire or lack the midbody-dependent abscission mechanism during their evolutionary history. However, the midbody is not essential for *Caenorhabditis elegans*, although it appears at the final abscission^27^. Unanchored human fibroblasts cannot complete cytokinesis even though they have a midbody^13^. In addition, recent studies have shown that some animal cells can also divide by contractile ring-independent cytokinesis^10,28–30^, suggesting that animal cells might have many modes of cytokinesis, similarly to *Dictyostelium* cells.

Previously, adhesions of *Dictyostelium* cells to the substratum coated with various materials have been investigated, showing that cell adhesion occurs on both hydrophilic and hydrophobic surface^31,32^. Zang *et al*.^6^ used a silane-based hydrophobic substratum and showed that wild-type cells but not myosin II-null cells can divide normally. We prepared non-adhesive substratum according to their reported method but found that the cells were mostly detached but sometimes adhered to the surface. Other previous experiments mimicking suspension conditions, i.e., *Dictyostelium* cells were suspended in the bottom of a droplet hanging from a coverslip^6^ or embedded in soft agar^33^, showed that wild-type cells but not myosin II-null cells can divide successfully. *Dictyostelium* cells as well as neutrophils can swim in viscous medium such as Ficoll^34^. Presumably, the matrix of soft agar or the high viscosity of Ficoll might provide some scaffolds for the cells to divide or swim. However, as observed in the present study, these cells could neither divide nor swim in low-viscosity medium without adhesion to the substratum. Interestingly, the cells can still extend pseudopods even though they cannot advance in the absence of scaffolds. It has become clear that cells can extend pseudopods without any scaffolds and cues, a topic that has been controversial^35^.

As mentioned in the Introduction, cell-substratum-adhesion mutants, such as talin-, vinculin-, sadA- and paxillin-null *Dictyostelium* cells, showed defects in cytokinesis. It is highly likely that they have defects in cytokinesis for the same reason as wild type cells on Lipidure-coated substratum.

On the substratum, in addition to constriction of the contractile ring, wild-type cells may require a traction force against the substratum for the final separation, similarly to the process of cytokinesis B. MiDASes (mitosis-specific dynamic actin structures), which appear underneath two daughter nuclei in dividing cells, play an important role in cell-substratum adhesion, particularly in cytokinesis B. However, MiDASes appear within a short time, even in wild-type cells^36^, indicating that wild-type cells simultaneously need and use cytokinesis B (traction force mechanism). Furthermore, traction force microscopy has shown that wild-type cells exert a traction force in the inward direction from the poles^37,38^. In suspension culture, we found that cells require an external shearing force introduced by shaking for the final separation. Otherwise, these cells need to form multicellular aggregates to complete cell division.

What is the mechanism of cytokinesis in the multicellular aggregates identified in the present study? Because this mode of cytokinesis depends on myosin II, we can exclude the hypothesis that cells divide by exerting a traction force against the surface of neighbor cells instead of the substratum in a similar manner to that found in cytokinesis B and C because these modes are independent of myosin II. However, at present, there still remains a possibility that this traction force-mediated mechanism is not sufficient but partially contributes to the cytokinesis in the multicellular aggregate. Cytokinesis D is also excluded because the chemotaxis-deficient mutant exhibited normal division in the aggregates. The midwife mechanism might still function without chemotaxis because the random movements of neighbor cells might accidentally aid the final abscission of the thin connection between the two daughter cells in a crowded and compacted multicellular aggregate, but this mechanism might be too unreliable and inefficient for the division of almost all of the cells in the aggregates. Together, the cells must divide through an additional and novel mode of cytokinesis in the aggregates.

The dividing cells could not complete cytokinesis by adhering to only a single cell (Fig. 4A). Instead, to complete cell division, at least 9–10 cells in the aggregates are required. Therefore, we propose that cells divide using friction in the confined environment found in multicellular aggregates as they divide in a confined space between two coverslips. To utilize friction in a confined space, the dividing cells have to move inside the aggregate. Because our observations show that the cells always change their position in the aggregate, the dividing cells may move inside the aggregates by chance or through an unknown mechanism.

The novel mode of cytokinesis requires myosin II. The results of our experiments show that wild-type cells can divide in aggregates composed of HS1 cells and that myosin II is required in the dividing cells but not in the peripheral cells (Fig. 4D). Pressing of the cells with an agar block resulted in increased accumulation of myosin II at the cleavage furrow than observed in the absence of the agar block^39,40^. Myosin II might contribute to the exertion of more force for efficient cytokinesis by accumulating at a higher level at the cleavage furrow. However, there was no apparent higher level of accumulation of myosin II at the cleavage furrow in confined conditions compared with normal conditions (Fig. 5E). Myosin II may accumulate under a constant pressure of agar-overlay but not in the confined conditions, where the height of the space is fixed. As another explanation of the contribution of myosin II to the cell division in the aggregate, myosin II might contribute to the friction-mediated movements of the daughter cells in opposite directions. The observation that after completing cell division, each daughter cell can migrate in the confined space (Fig. 5B) supports this idea. Further experiments are needed to clarify the molecular mechanism of friction-mediated cytokinesis, and investigate the role of myosin II on it.

Some animal cells are able to make multicellular aggregates (spheroids) in suspension culture. Spheroids are used as biological models of native tissue, and the environment within spheroids is considered a more physiological condition. The cells can divide and multiply in spheroids, but there is no information regarding the mode of cytokinesis discussed in this study. Recent studies have shown that cell division in tissue is very different from that which occurs in the 2D condition. In the *Drosophila* pupal notum, neighboring interphase cells are pulled by the formation of the furrow of the dividing cell, and myosin II accumulates near the cleavage furrow in the neighbor cells to aid the process of cytokinesis^41^. However, our preliminary observations did not show such specific accumulation of myosin II in neighboring *Dictyostelium* cells, which is consistent with that neighboring cells do not need myosin II for the dividing cell to complete the abscission (Fig. 4D).

**Figure 7:**
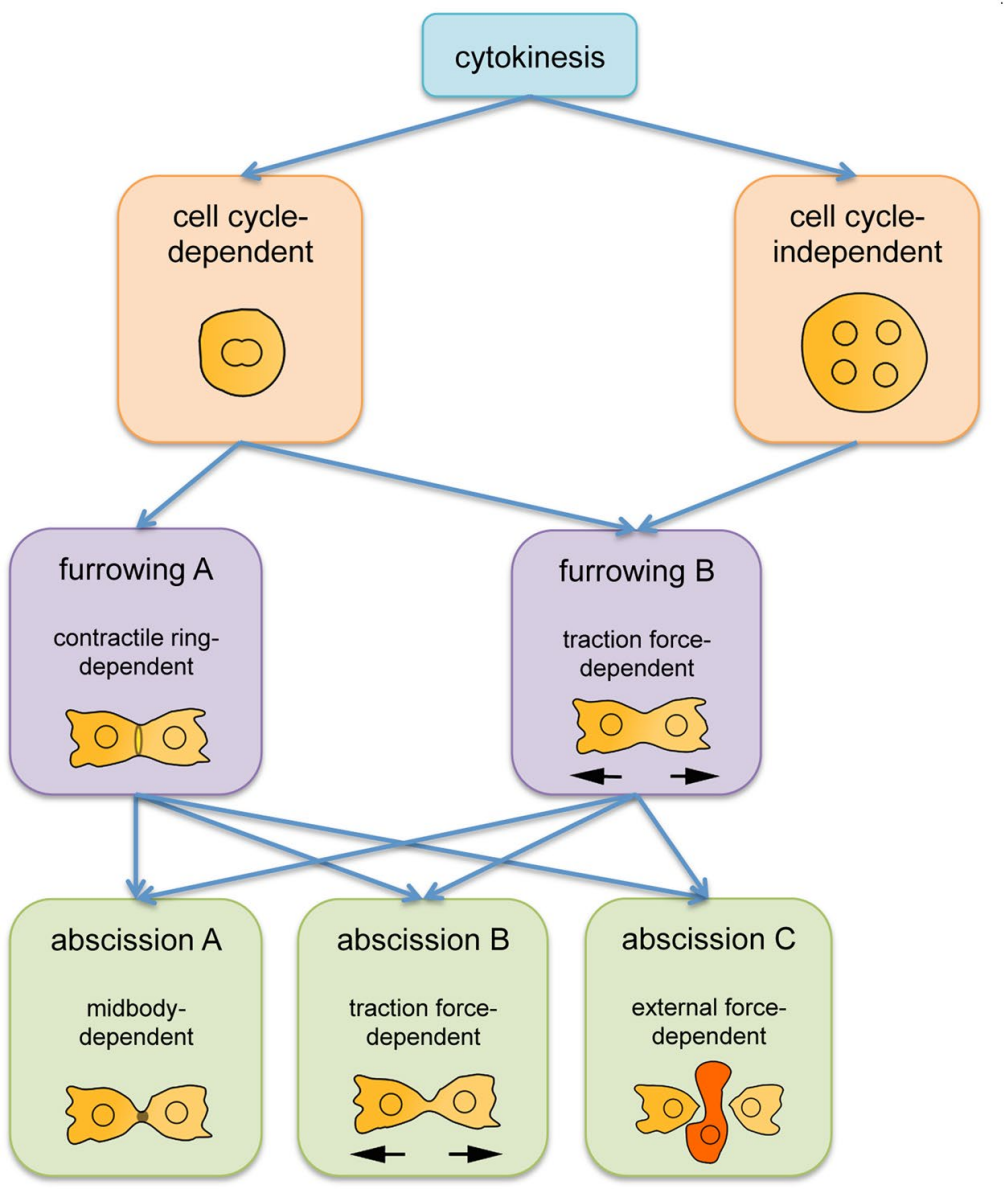
Classification of multiple modes of cytokinesis. There are two main categories: cell cycle-dependent and cell cycle-independent cytokinesis. The conventional process of cytokinesis C belongs to the latter. The furrowing step can be classified into two classes: contractile ring-dependent (furrowing A) and contractile ring-independent (furrowing B). The abscission step can be classified into three classes: midbody-dependent (abscission A), traction force-dependent (abscission B), and external force-dependent (abscission C). Most animal cells divide by furrowing A and abscission A. Wild-type *Dictyostelium* cells divide on the substratum by furrowing A and abscission B but divide in suspension by furrowing A and abscission C (with the shearing force of shaking as the external force). Myosin II-null cells divide on the substratum by furrowing B and abscission B. The friction-mediated process of cytokinesis observed in the present study might be classified as contractile ring-dependent (furrowing A) and traction force-dependent (abscission B). The cells dividing via conventional cytokinesis D (midwife-dependent cytokinesis) divide by furrowing A (or B) and abscission C (with a help of the external force provided by the midwife cell).

We thus propose to redefine and classify multiple modes of cytokinesis for animal-type cytokinesis (Fig. 7). Although there are some common features between animal and plant cytokinesis, such as midbodies and phragmoplasts, which are analogous structures involved in cytokinesis^42,43^, we want to focus on animal-type cytokinesis. There are two main categories; cell cycle-dependent and cell cycle-independent cytokinesis. The conventional cytokinesis C belongs to the latter. Recent studies have suggested that there are two steps in cytokinesis, specifically furrowing and abscission steps^44,45^. The furrowing step can be classified into two classes: contractile ring-dependent (furrowing A) and contractile ring-independent (furrowing B). In addition, the abscission step can be classified into three classes: midbody-dependent (abscission A), traction force-dependent (abscission B), and external force-dependent (abscission C). The friction-mediated cytokinesis observed in the present study might be classified as traction force-dependent (abscission B), because the cell divides by traction force in a confined space, although they have no specific anchorage. The cells dividing by conventional cytokinesis D (midwife-dependent cytokinesis) divide by furrowing A (or B) and abscission C (with help from an external force provided by the midwife cell). Based on this classification, most animal cells divide by furrowing A and abscission A. Wild-type *Dictyostelium* cells divide on the substratum by furrowing A and abscission B but divide in suspension by furrowing A and abscission C (with the shearing force of shaking as the external force). Myosin-null cells divide on the substratum by furrowing B and abscission B.

What is the physiological role of the formation of aggregates of *Dictyostelium* cells? The plasticity of the division mode might be important for cell survival under various environmental conditions. *Dictyostelium* cells live in the interface of soil and air and might often lose their substratum; thus, it is plausible that these cells can form multicellular aggregates to divide through the alternative cytokinesis method to survive under such a severe environment.

## Methods

### Cell culture

*Dictyostelium discoideum* wild-type (AX2), myosin II-null (HS1), csB-null, and Gß null cells were cultured at 22 °C in plastic dishes containing HL5 medium (1.3% bacteriological peptone, 0.75% yeast extract, 85.5 mM D-glucose, 3.5 mM Na_2_HPO_4_^.^12 H_2_O, and 3.5 mM KH_2_PO_4_, pH 6.3). An extrachromosomal vector for the expression of EGFP-histone or EGFP-myosin II was introduced into the cells by electroporation, as described previously^46^. The transformed cells were selected in HL5 medium containing 10 µg/ml blasticidin (Kaken) in plastic dishes. For shaking culture, the cells were cultured in conical flasks (100 ml) containing 20 ml of HL5 medium in a reciprocal shaker (stroke width, 2 cm) at 150 rpm unless otherwise noted.

### Non-adhesive coating

Coverslips and plastic dishes were coated with Lipidure, a water-soluble polymer of 2-methacryloyloxy ethyl phosphorylcholine (CM5206, NOF, Japan). Ten microliter of 0.5% (w/v) Lipidure (dissolved in ethanol) was spread on the surface of a coverslip and air-dried at 22 °C. Plastic dishes (5.5 cm in diameter) were coated by pouring 1 ml of Lipidure, removing the excess solution and then air-dried. A solution of 2% (w/v) poly 2-hydroxyethyl methacrylate (poly-HEMA, P3932, Sigma) dissolved in ethanol was also used for coating using the same procedure.

### DAPI staining

Cells were collected by centrifugation at 2,000 rpm for 2 min. The cell pellet was mixed with a fixative solution (2.5% formalin and 15 mM Na/K phosphate buffer, pH 6.3) for 10 min. Four microliters of the fixed cell suspension were mixed with 2 µl of 4 µg/ml 4′,6-diamidino-2-phenylindole (DAPI, Sigma) on glass slides. The cells were covered with a coverslip and then pressed using filter paper and fingers. The cells that contained two or more nuclei were considered multinucleated cells.

### Medium containing methyl cellulose

To prevent adherence of the cells to each other to form aggregates, the cells were cultured in HL5 medium containing 1% (w/v) methyl cellulose (M-0512, Sigma). After the methyl cellulose was dissolved in HL5 medium using a stirrer at 80 °C, the solution was autoclaved.

### Microscopy

The cells were placed in Lipidure-coated or non-coated glass bottom dishes and observed under an inverted differential interference-contrast microscope (DIC) or a phase-contrast microscope (Olympus, IX71). To visualize the nucleus, DAPI-stained cells were observed under a fluorescence microscope (Nikon, TMD300) by UV illumination^47^. The cells expressing GFP-histone or GFP-myosin II were observed with a DeltaVision microscope system based on an Olympus IX71 inverted microscope (GE Healthcare Life Sciences, Japan) with a 60× objective lens (PLAPON 60× OTIRFM, NA 1.45). Fluorescence images were acquired by z-sectioning at 1-μm intervals. The exposure times for GFP and DIC were set to 0.1 and 0.05 s, respectively.

To observe the cells placed in a confined space, cells and polystyrene beads (3, 4.5, 6, or 10 µm in diameter, PolyScience) were mixed, placed on a Lipidure-coated coverslip (24 × 40 mm), and overlaid with another Lipidure-coated coverslip (18 × 18 mm). Excess medium was removed using a filter paper from the edges of the coverslip. When the Brownian movement of the beads was stopped as the medium was removed, the width was considered to be set to the specified diameter of the beads.

### Image processing

Deconvolution of three-dimensional z-stacks was performed with softWoRx (GE Healthcare Life Sciences, Japan). The deconvoluted images were further processed using Volocity software (Improvision) and ImageJ (http://rsb.info.nih.gov/ij).

### Statistical analysis

Statistical analysis was performed using GraphPad Prism 7 (GraphPad Inc, USA).

Data are presented as mean ± SD and analyzed by one way ANOVA with Tukey’s multiple comparison test.

### Data availability

All relevant data are available from the authors on reasonable request.

## Electronic supplementary material

Supplementary Information

A typical time course of formation of multicellular aggregates in detached condition
