# Supplementary material for: A novel mode of cytokinesis without cell-substratum adhesion
Source: Sci Rep. 2017 Dec 18;7:17694. doi: 10.1038/s41598-017-17477-w (PMC5735089; doi:10.1038/s41598-017-17477-w)
Supplement: Supplementary file 1 — Supplementary Information [file 41598_2017_17477_MOESM1_ESM.pdf]

Supplementary Information for

A novel mode of cytokinesis without cell-substratum adhesion

Risa Taira and Shigehiko Yumura

Supplementary Figure S1

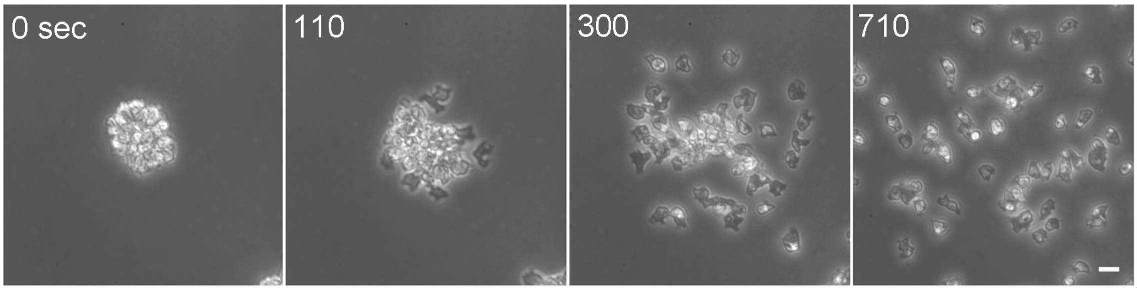

Supplementary Figure S1 Placement of the multicellular aggregate on a non-coated coverslip resulted in an immediate dispersion of the aggregate

When the multicellular aggregates were placed on a non-coated coverslip, they dispersed to single migrating cells within approximately 10 min. Bar, 10 μm.

Supplementary Figure S2

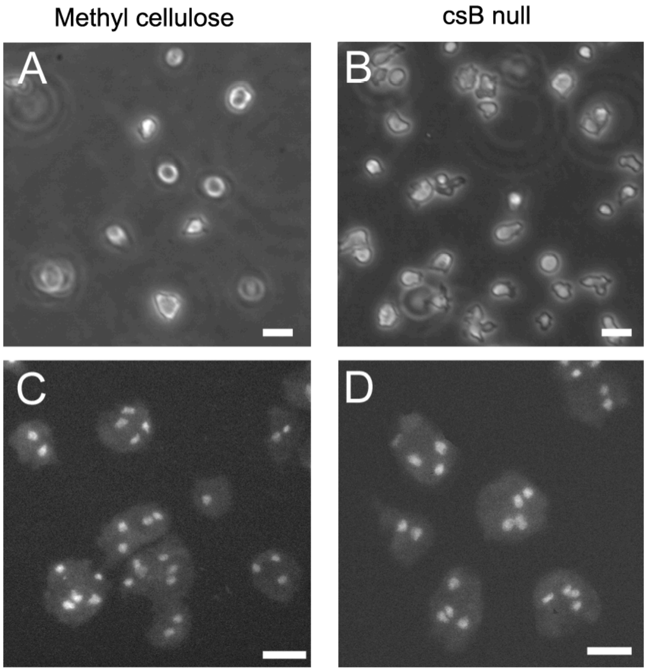

Supplementary Figure S2 Cells became multinucleated when detached cells were cultured separately

(A) Wild-type cells were cultured in a Lipidure-coated dish with HL5 medium containing 1% methyl cellulose (MC) for 24 h. (B) Mutant cells deficient in three genes encoding cell-cell adhesion contact site B (csB) proteins (csbA, csbB, and csbC) were cultured in a Lipidure-coated dish. The formation of aggregates was significantly suppressed, although some small aggregates could be observed. (C and D) Fluorescence images of DAPI-stained cells 24 h after culturing as described above. The cells became significantly multinucleated: in the presence of MC (C) and csB-triple-mutant cells (D). Bars, 10 μm.

Supplementary Figure S3

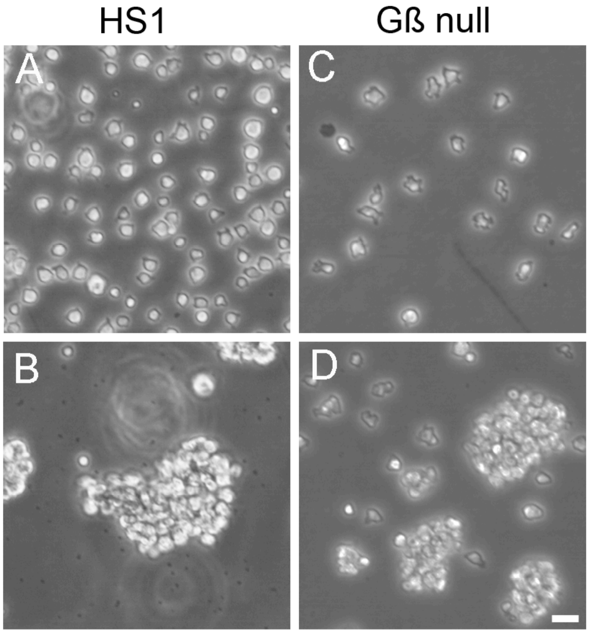

Supplementary Figure S3 Myosin II-null cells and Gß null cells can form multicellular aggregates

(A and B) Myosin II-null (HS1) cells were cultured for 24 h in a Lipidure-coated culture dish for (A) 0 h and (B) 24 h and formed multicellular aggregates after 24 h. (C and D) Gß-null cells were cultured for 0 h (C) and 24 h (D) in a Lipidure-coated culture dish and formed multicellular aggregates, even though some single cells remained. Bar, 10 µm.

Supplementary Video legend

Supplementary Video S1
A typical time course of formation of multicellular aggregates in detached condition

When wild type cells were placed in a Lipidure-coated plastic dish, they could not attach to the bottom surface of dish. The cells gradually adhered to each other, resulting in the formation of multicellular aggregates.
